# Superior linear and comparable rotational protection of an air-filled helmet versus foam helmets

**DOI:** 10.1038/s41598-025-10615-9

**Published:** 2025-07-27

**Authors:** R. Tan, C. E. Baker, X. Yu, M. Ghajari

**Affiliations:** 1 HEAD Lab, Dyson School of Design Engineering, Imperial College, London, SW7 2AZ UK; 2 Department of Mechanical Engineering, University of Sheffield, Sheffield, S10 2TN UK

**Keywords:** Biomedical engineering, Mechanical engineering

## Abstract

Air-filled chambers offer a promising approach for designing lightweight and portable bicycle helmets, yet their effectiveness in real-world cycling accidents, particularly under oblique impacts, remains unexplored. Here, for the first time, we evaluated the brain injury mitigation performance of a commercially available air-filled helmet, Ventete aH-1, under oblique impacts, and compared it with three conventional cycle helmets, ranking high, middle and low in a recent study of 30 cycle helmets. Helmets were fitted to a new headform with more biofidelic physical properties than other existing headforms, allowing for more accurate measurements of linear and rotational motion during impacts. The helmeted headform was subjected to impacts to the front, front-side, side and rear against a 45° anvil at 6.5 m/s. The risk of linear and rotational injuries was calculated using risk functions based on PLA (peak linear acceleration) and BrIC (brain injury criterion) and exposure weighting. The PLA and linear risk were lower for the air-filled helmet than the EPS helmets in all impact locations. The air-filled helmet showed a 44% reduction in overall linear brain injury risk compared to the best-performing EPS helmet, attributed to its nearly twice as long impact duration. The air-filled helmet’s rotational performance compared to the EPS helmets was dependent on the impact location, with its overall rotational risk being slightly better than the EPS helmet ranked middle. Our study shows that air-filled chambers have the potential to provide superior protection compared with EPS liner helmets under oblique impacts. We hope our results will inspire new helmet designs which adopt air-filled chambers to improve brain injury protection and address portability concerns that limit helmet adoption.

**Supplementary Information:**

The online version contains supplementary material available at 10.1038/s41598-025-10615-9.

## Introduction

Cycling has significant health and environmental benefits, and it is growing in its popularity particularly in large cities^1^. However, as cycling usage increases, the number of individuals killed or seriously injured also increases^2^. It is reported that globally, road injury is the leading cause of death for those aged 5–29, of which there are 70,000 cyclist fatalities each year^3^. Traumatic brain injury (TBI) is the primary cause of fatalities among cyclists. Where incidents cannot be prevented, the use of helmets remains the most effective defence against TBI. Multiple studies have shown that cyclists who wear helmets are protected from TBI of all severities, especially skull fractures and subdural hematoma^4–6^.

Despite the clear safety benefits, helmet adoption rates remain low^7^. Studies examining helmet usage among 2,424 cyclists aged 7 to 59 years old and focus groups involving parents and children identified several factors contributing to low helmet usage^8–10^. These include confusion about effectiveness and cost of helmets as well as concerns about helmet storage, comfort and aesthetics. These challenges have complicated efforts to redesign conventional Expanded Polystyrene (EPS) helmets to better align with consumer preferences, as there are constraints related to maintaining the necessary thickness and stiffness of the EPS liner to ensure adequate protection^11^. Therefore, there is a growing interest in exploring alternative energy-absorbing materials that offer greater design flexibility.

Several novel materials have been explored for helmet application including thermoplastics^12^, hybrid composites^13^, collapsible cellular structures^14^, bio-inspired materials such as pomelo fruit^15^, viscoelastic cellular cells^16^ and several other structures^17–19^. While these materials demonstrate promising energy absorption properties, their high production costs, durability, and manufacturability have largely hindered commercial adoption.

An alternative approach is air-based impact mitigation, a concept used in airbags and rubber fenders to increase the contact time, spreading the contact force over a longer duration. This improves crash survivability^20–22^. Air-based impact absorption has been explored in American football, motorcycle, construction, and even military helmet applications^21–24^. This technology has also been integrated into bicycle helmets, with examples such as Hövding 3.0 and Bumpair helmets, which both demonstrated a significant reduction in the head peak linear acceleration (PLA) compared with conventional EPS helmets^20,24,25^. However, these innovations face challenges. Despite leading in air technology for bicycle helmets since 2011, Hövding, an airbag helmet worn around the neck and inflating during accidents, filed for bankruptcy in 2023. This was due to a sales ban prompted by safety concerns related to deployment failure. As a result, there is significant anticipation for more conservative air-filled helmets that remain on the head, like conventional EPS helmets.

While air-filled bicycle helmets have been explored in a few studies^6,26^, there is a critical gap in understanding their performance under oblique impacts, the most common type of collision in real-world cycling incidents, often resulting in large head rotation. Existing research on air-filled bicycle helmets has primarily focused on linear impact mitigation, leaving their effectiveness against rotational injuries, a major contributor to diffuse brain injuries, largely unexplored^6,26^. To date, the Hövding helmet appears to be the only air-filled helmet assessed under oblique impact conditions^27^. This study showed that Hövding helmet had superior performance in reducing head linear and rotational accelerations compared with EPS helmets, highlighting the potential of air-based technologies in mitigating head injuries. However, the rotational injury mitigation performance of other air-filled cycle helmets, particularly those with air-filled chambers, remains unexplored.

In this study, we address this gap by evaluating the safety performance of a commercially available air-filled chamber helmet under oblique impact conditions. We assess its ability to reduce both linear and rotational head motion, key indicators of skull fracture and diffuse brain injuries, respectively. The performance of the air-filled helmet is compared with three conventional EPS-liner helmets selected from a comprehensive study of 30 cycling helmets^28^. We investigate whether the air-filled helmet can outperform these conventional EPS helmets in mitigating linear and rotational brain injuries and explore its protective mechanisms. This study provides new insights into the potential of novel air-filled helmet technologies for brain injury prevention under oblique impacts.

## Materials and methods

### The air-filled helmet

**Fig. 1:**
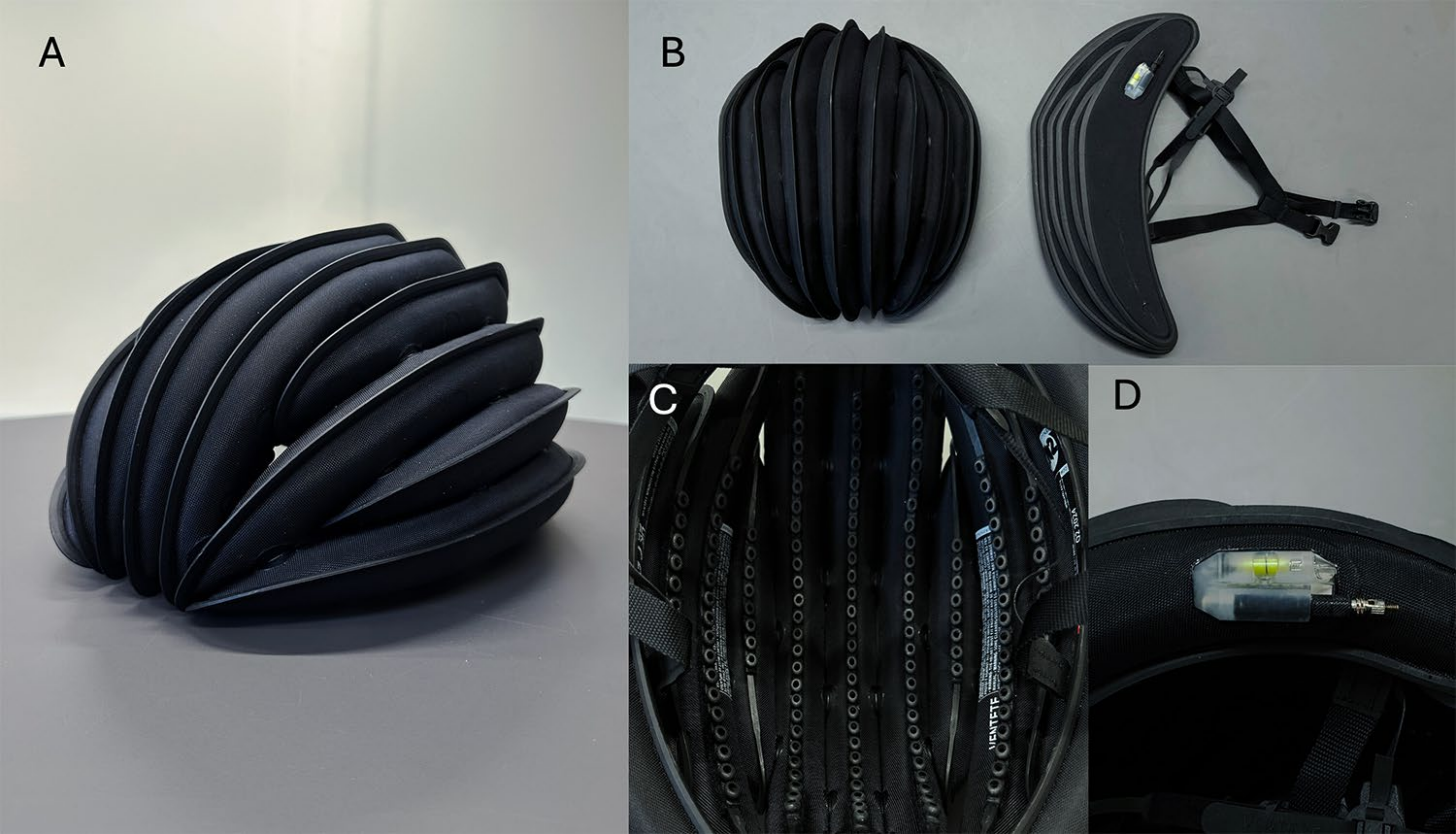
(**a**) The air-filled VENTETE helmet comprised of 11 interconnected chambers. (**b**) The inflated and deflated shapes. (**c**) The Rheon internal lining. (**d**) The valve and integrated pressure indicator.

The air-filled helmet investigated in this study is sold under the commercial name VENTETE (model aH-1 R). It is an inflatable, collapsible helmet. According to the manufacturer, it is designed for use with bicycles, e-bikes, scooters, e-scooters, skateboards and roller-skates. When deflated, it is less than 4 cm thick to enable storage. The deflated volume of the helmet is approximately 10% of its inflated volume (Fig. 1). It has passed the EN1078 standard tests, and as such it holds a CE certification.

The helmet body consists of a series of 11 interconnected, inflatable chambers. Each chamber is encased in a protective rib. The chambers are built of a laminated nylon, which is puncture, abrasion and stretch resistant. The external ribs, moulded from glass-reinforced polymer, provide additional structural rigidity.

An internal lining is integrated with the ribs. It is made of an elastomer with energy absorbing properties, sold under the commercial name Rheon^29^. This liner was added to the helmet to improve comfort and potentially provide better mitigation of head rotation.

The helmet is inflated via a valve, which includes an integrated pressure indicator that displays the internal pressure. The helmet is inflated to 32 psi, with an approximate helmet volume of 1.5 L. The mass of the medium size helmet is 465 g.

### The EPS-liner helmets

To compare the performance of the air-filled helmet with EPS-liner helmets, we tested three bicycle helmets, Specialized Align II MIPS, Bontrager Solstice and Halfords Urban, which all have an EPS liner. These helmets were selected from a recent study of 30 popular helmets sold in the UK^28^. This study showed significant variations in head kinematics across different helmet models, even though all helmets met the EN1078 standard. Rotational risks showed a greater variation than linear risks. For this study, we selected helmets from three different performance categories: high (rank 2), middle (rank 16), and low (rank 30).

### Experimental setup

**Fig. 2:**
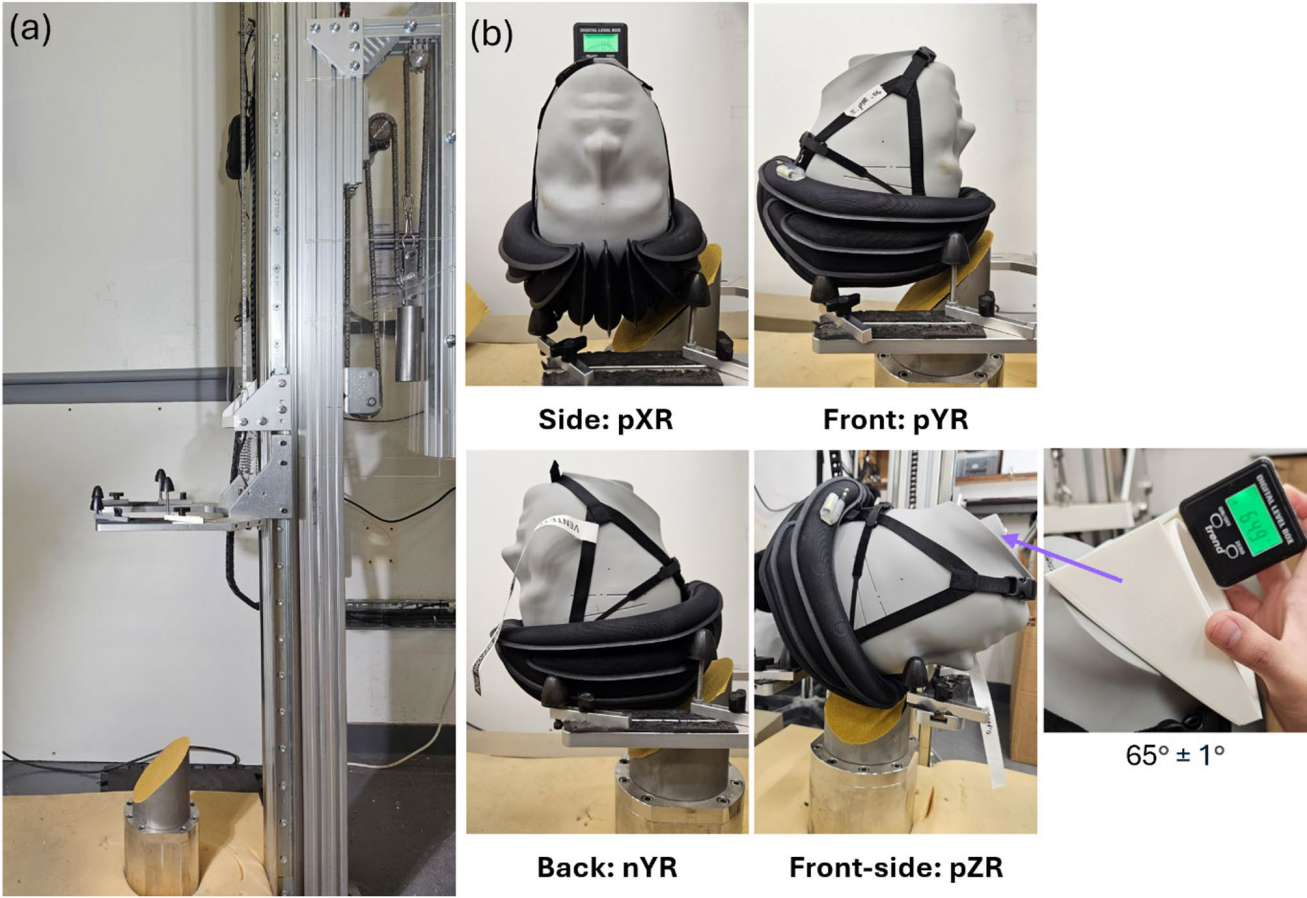
(**a**) The drop tower test rig. (**b**) The four tested impact locations. Here X, Y and Z refer to the axis of the dominant rotational velocity component and R refers to rotation. The direction of rotation is indicated by p (positive) and n (negative).

The helmets were evaluated according to the test protocol described in detail in^28^. The helmets were exposed to a series of four oblique impacts to the front, rear, side and front-side of the helmet at an impact speed of 6.5 m/s and an impact angle of 45°. These impact configurations produce dominant rotation about one of the anatomical locations of the head. We use this axis to refer to the impacts, i.e. side impact: pXR, front impact: pYR, rear impact: nYR and front-side impact: pZR (Fig. 2b). The impact surface was covered with a 80 grit abrasive paper. These impact conditions are guided by representative real-world cycling accidents, as supported by a comprehensive literature review on cyclist head injuries and impact characteristics^6^. These conditions have also been used in multiple studies to assess bicycle helmets^20,30,31^.

The tests were conducted with the drop tower test rig at the Human Experience, Analysis and Design (HEAD) Lab, Imperial College, at room temperature of 18–20 °C. This rig employs a free-fall drop and features a U-shaped testing platform for guiding the helmeted headform during the fall (Fig. 2a). The helmets were fitted on the headform with a consistent 27.5 mm distance between the helmet edge and the top edge of the orbital mark on the headform. The chin strap was secured to a standard tightness, leaving approximately 10 mm of space under the chin (a ‘finger space’) before positioning on the testing platform. A digital inclinometer ensured the headform was precisely adjusted. Specifically, 0° ± 1° inclination was maintained for pXR, pYR, and nYR impacts, while pZR was set at 65°± 1° (Fig. 2). A high-speed camera positioned behind the anvil captured impact footage 0.5 s before and after impact at a 3500 frame per second rate.

Each impact was repeated three times. For each impact, a fresh air-filled helmet was used; in total, 12 air-filled helmets underwent testing across all four locations. Before every impact, the helmet was pumped to the pressure defined by the manufacturer. Each EPS helmet was subjected to impacts at two different locations: pXR and pYR, or nYR and pZR. Hence, a total of six helmets were used to test each EPS helmet model.

### The headform

We used a new headform designed according to the EN17950 standard^32^. The specifications of this headform were defined by the European Standardisation head protection Committee (CEN/TC158/WG11) to better represent shape, mass, mass moments of inertia (MoI) and coefficient of friction (CoF) of the human head^33,34^. We used a 570 mm CEN headform manufactured by Cellbond. We previously used an older version of this headform for testing 30 bicycle helmets^28^. The key improvement of the new version of this headform is the CoF, which is closer to the standard range. The physical properties of this version of the Cellbond CEN headform are provided in Table 1.

**Table 1:** Physical properties of cellbond CEN headform used in this study.

Circumference [mm]	Mass [kg]	MoI xx [kg.cm^2^] ^1^	MoI yy [kg.cm^2^] ^1^	MoI zz [kg.cm^2^] ^1^	CoF ^2^
570	4.27	214.40	243.67	149.14	0.33 ± 0.01

^1^MoI: Moment of Inertia. Measurements obtained from the CAD model of the headform.

^2^CoF: Coefficient of Friction. Measured in the HEADLab, Imperial College London, on 21st June 2024, using the EN17950 friction testing apparatus.

The headform was fitted with the DTS 6DX PRO sensor package, which was operated by a wireless data logger system, to measure linear accelerations and rotational velocities along the x, y, and z axes. The linear accelerometers were placed at the centre of gravity (CoG) of the headform, measuring the linear acceleration of headform’s CoG. Data was collected 0.5 s before and after impact at a sampling rate of 20 kHz. Linear accelerations and rotational velocities were filtered using CFC600 and CFC180, respectively, in accordance with ISO 6487 standards. These filtered signals were then used to compute resultant values. As the study did not involve human or animal subjects, formal ethical approval was not required.

### Injury assessment

Helmeted cyclists involved in falls and collisions can sustain different types of brain injuries, which can be placed under two main categories, focal and diffused^28,35–37^. Different metrics have been developed to predict these injuries. In this study, we used PLA and Brain Injury Criterion (BrIC). PLA is directly related to the force exerted on the head according to Newton’s second law of motion and can be used to predict skull fractures, associated bleeding and contusions^38,39^. Conversely, BrIC, which uses the rotational velocity of the head, predicts the risk of diffuse brain injuries^40–42^.

To assess the safety efficacy of the air-filled and EPS helmets, we used risk functions for linear and rotational brain injuries, which were used in our previous study of 30 cycle helmets^28^. The following injury risk function based on PLA was used to evaluate the performance of the helmet in preventing focal injuries:

1 $$\:P\left(linear\right)=\frac{1}{\left[1+{e}^{\left(3.3202-0.01312\text{*}PLA\right)}\right]}$$

where

2 $$\:PLA=\text{m}\text{a}\text{x}\left(\sqrt{{{a}_{x}\left(t\right)}^{2}+{{a}_{y}\left(t\right)}^{2}+{{a}_{z}\left(t\right)}^{2}}\right)$$

$\:{a}_{x}\left(t\right)$, $\:{a}_{y}\left(t\right)$ and $\:{a}_{z}\left(t\right)$ represent the components of the head linear acceleration measured at the head CoG at the same time instance. Equation (1) is based on the risk function presented for older adults^43^, which was modified to represent the general population^28^. To assess the helmets’ ability to prevent diffuse brain injuries, the following injury risk function was used^41^:

3 $$\:P\left(rotational\right)=1-{e}^{-{\left(\frac{BrIC}{0.602}\right)}^{2.84}}$$

where

4 $$\:BrIC=\sqrt{{\left(\frac{max\left(\right|{\omega\:}_{x}\left(t\right)\left|\right)}{{\omega\:}_{xC}}\right)}^{2}+{\left(\frac{max\left(\right|{\omega\:}_{y}\left(t\right)\left|\right)}{{\omega\:}_{yC}}\right)}^{2}+{\left(\frac{max\left(\right|{\omega\:}_{z}\left(t\right)\left|\right)}{{\omega\:}_{zC}}\right)}^{2}.}$$

$\:{\omega\:}_{x}\left(t\right)$, $\:{\omega\:}_{y}\left(t\right)$ and $\:{\omega\:}_{z}\left(t\right)$ are the components of the head rotational velocity and $\:{\omega\:}_{xC}$ = 66.25 rad/s, $\:{\omega\:}_{yC}$ = 56.45 rad/s and $\:{\omega\:}_{zC}$ = 42.87 rad/s.

Linear and rotational injury risks were calculated for each impact location and their average was calculated to indicate the overall injury risk for each impact location. The total overall injury risk for the helmet was calculated by multiplying the risk for each impact location by exposure probability and summing the weighted risks. A previous study determined the exposure probabilities from a meta-analysis of 1,809 impacts, as pXR​ (side): 0.287; pYR​ and pZR​ (front): 0.191; nYR​ (rear): 0.203^6^.

### Statistical analysis

We calculated the mean, standard deviation and coefficient of variation (CV) across all repetitions for each helmet (*n* = 3 per helmet). We used the z-score to determine if a significant difference existed in head kinematics between a conventional EPS helmet and the air-filled helmet^20^. For a two-side t-test and a significance level of 0.05, a z-score outside the range of − 1.96 to + 1.96 indicates a significant difference.

## Results

### Consistent peak kinematics values across impact repeats

The coefficient of variation (CV) of peak head kinematics across impact repeats was small for the air-filled helmet (Appendix 2). The CV for PLA was 3–8%, for PRV (Peak Rotational Velocity) it was 2–8% and for BrIC it was 2–8%, depending on the impact location. The CV was higher for PRA (Peak Rotational Acceleration), 11–14%. The CV for the EPS helmets were similar, and in some cases, it was higher than the air-filled helmet. The range of peak kinematics CVs for the EPS helmets was 2–5% for PLA, 1–11% for PRV, 1–11% for BrIC and 1–19% for PRA. Overall, these results show good consistency of the peak kinematics values across impact repeats for most impacts locations and helmets.

### The air-filled helmet prolongs the impact

**Fig. 3:**
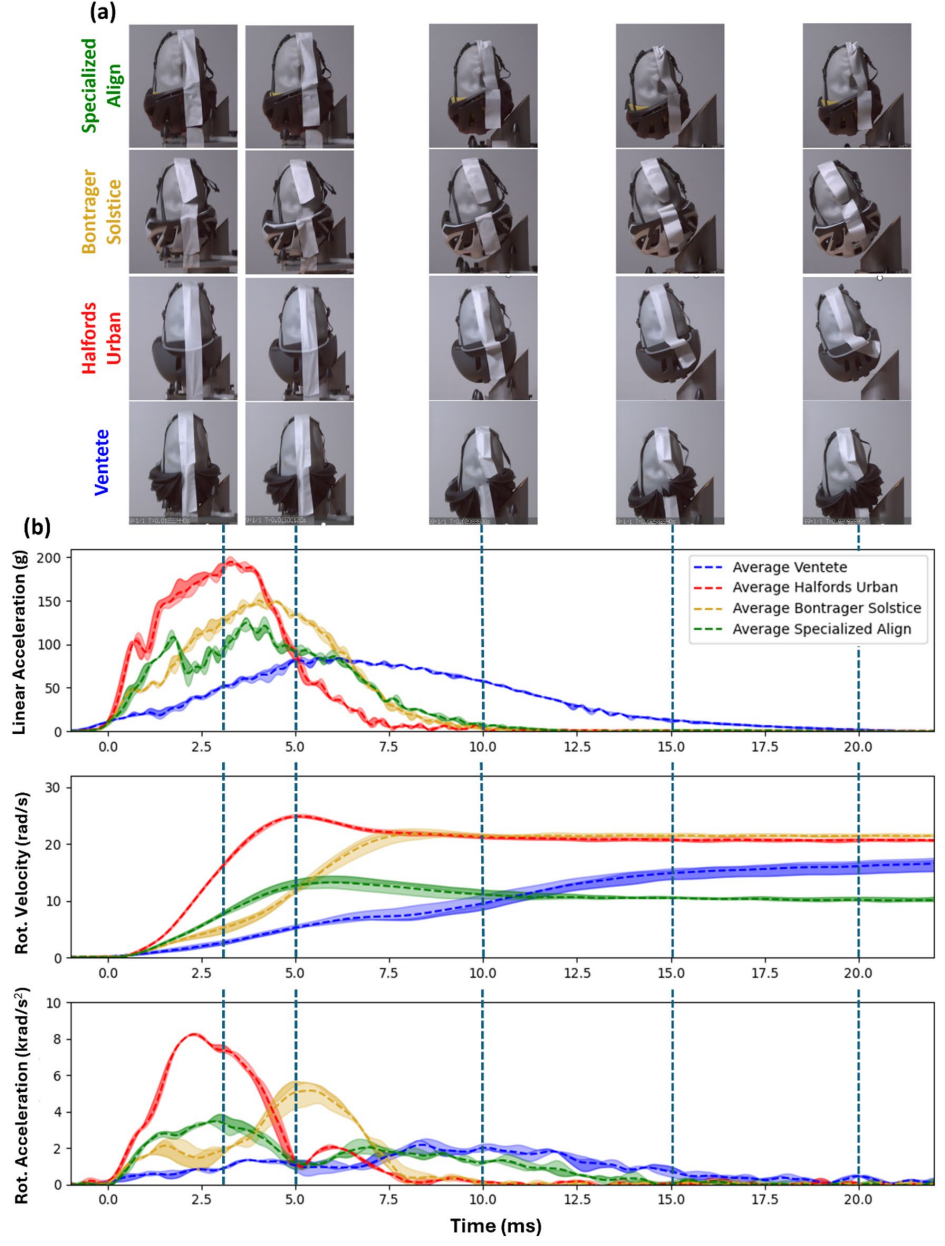
The results for the VENTETE, Specialized Align MIPS, Bontrager Solstice and Halfords Urban helmets. (**a**) Snapshots from high-speed videos of helmets taken between 3 ms and 20 ms after the impact at location pXR. These snapshots capture the entire impact duration as represented in the corresponding time-history graph. (**b**) The dotted line represents the average resultant linear acceleration, rotational acceleration, and rotational velocity for each tested helmet. The colour-filled area represents the range between the maximum and minimum values across the three repetitions of each helmet.

The air-filled helmet remains in contact with the anvil for longer than the EPS helmets, as can be seen in Fig. 3(a), which shows the pXR impact (other impact locations can be seen in Appendix 1). The impact duration for both linear and rotational accelerations is nearly twice as long for the air-filled helmet than the EPS helmets (Fig. 3b). The EPS helmets have an impact duration of up to 10 ms, with peaks occurring between 3 and 5 ms. The air-filled helmet extends this duration to 20 ms, with a peak between 6 and 8 ms. This figure also shows noticeable relative rotation between head and helmet for the Specialized Align MIPS and air-filled helmet, leading to small head rotation even at 20 ms.

### Peak linear acceleration is lower for the air-filled helmet

**Fig. 4:**
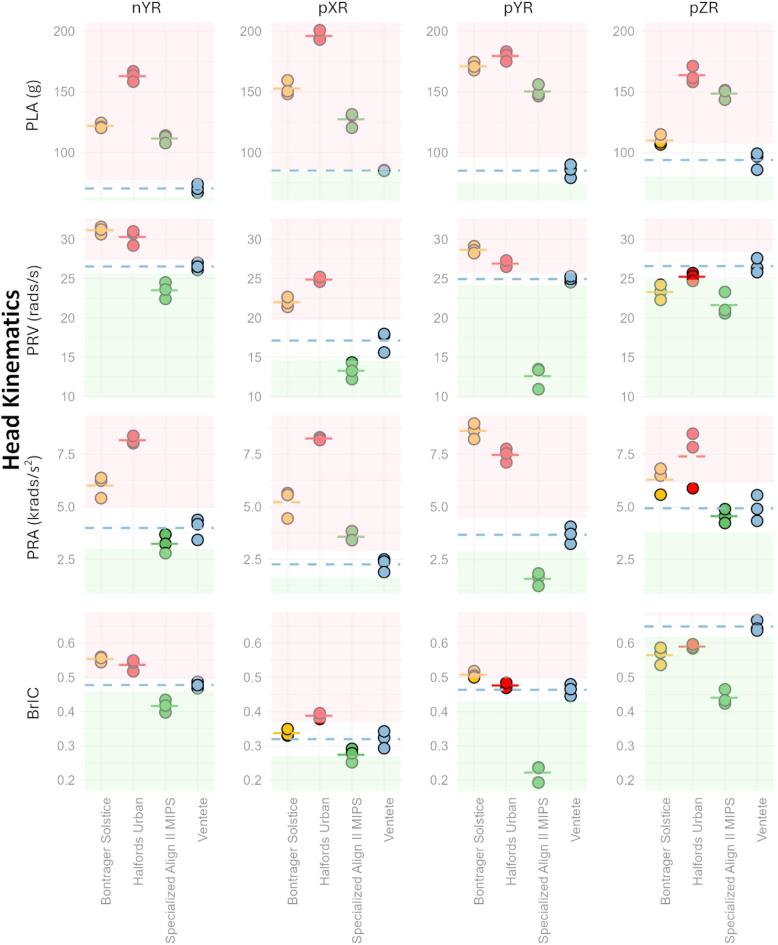
Head kinematics (PLA, PRA, PRV, and BrIC) were evaluated for all helmets at impact locations nYR, pXR, pYR, and pZR. Each helmet model is distinguished by a unique colour, with the dotted line representing the average metric value for that helmet. The average value for the VENTETE helmet is extended across all models in blue dotted line. Light pink and green shaded areas indicate helmets that perform significantly worse or better than the air-filled helmet, respectively, with significance level of 0.05, corresponding to a z-score beyond ± 1.96.

The air-filled helmet consistently produced lower PLA values across all impacts compared with the EPS helmets, with mean PLA reductions of nYR: 45–80%, pXR: 40–79%, pYR: 56–72% and pZR: 16–54% (Fig. 4).

### Rotational performance of the air-filled helmet is comparable with the EPS helmets without MIPS

The air-filled helmet produced higher PRV than the MIPS-equipped helmet across all impact locations, with mean PRV increases of nYR: 12%, pXR: 25%, pYR: 66% and pZR: 21%. The BrIC values for the air-filled helmet were also higher in three locations, with mean BrIC increases of nYR: 14%, pYR: 70% and pZR: 38%. For the pXR impact, no significant difference was observed, though the air-filled helmet produced 15% higher mean BrIC than the MIPS helmet. There was no clear trend in PRA across all locations. While the air-filled helmet generally had a higher mean PRA compared with the MIPS helmet (nYR: 21%, pYR: 80%, pZR: 8%), the differences were not significant at locations nYR and pZR. At location pXR, the PRA for the air-filled helmet was significantly lower by 45%.

In comparison to the other two EPS helmets without dedicated anti-rotational systems, the air-filled helmet reduced the average PRA across all impact locations (nYR: 40–69%, pXR: 40–79%, pYR: 68–80%, pZR: 24–40%). A reduction in PRV was also observed in three locations (nYR: 13–16%, pXR: 25–37%, pYR: 8–14%). In pZR, PRV was significantly higher by 13%, with no significant difference compared to one of the helmets. BrIC values were generally lower, with significant reductions of 12–15% in location nYR compared with both EPS helmets. In locations pXR and pYR, BrIC values were reduced by 5–19% and 3–9%, respectively, though some reductions were not significant. However, at location pZR, the air-filled helmet showed 10–14% higher BrIC values compared to the two EPS helmets.

### Lowest risk of linear injuries with the air-filled helmet

The linear, rotational, and overall risks of each helmet were calculated based on the measured kinematics and the probabilities of exposure across different impact locations (Table 2). Among the four helmets tested, the air-filled helmet had the lowest linear risk, which was driven by the lower PLA. This helmet had the second lowest rotational and overall risk, following the EPS helmet equipped with MIPS.

**Table 2:** The overall, linear and rotational risk of the helmets calculated with the weighted risk from all locations.

Helmet Model	Technology	Overall Risk	Linear Risk	Rotational Risk
Specialized Align II MIPS	EPS + MIPS	0.158	0.152	0.165
VENTETE	Air-filled chambers	0.209	0.085	0.334
Bontrager Solstice	EPS	0.261	0.165	0.357
Halfords Urban	EPS	0.303	0.238	0.369

## Discussion

Air-filled chambers increase portability of helmets, and our study shows their potential for providing linear protection better than conventional EPS helmets while providing rotational protection comparable with conventional EPS helmets. The superior reduction in the PLA experienced by the headform was achieved by almost doubling the impact duration compared with conventional helmets in all impact locations. This led to the best linear risk performance across all impact locations, with an average PLA of 84 g and a linear risk of 0.085, which was 44.1% lower than the best-performing EPS helmet in this study (PLA: 134 g; Linear risk: 0.152). No evidence of bottoming out was observed from the time history data, indicating that the helmet did not reach its deformation limit in any impact locations. It is noteworthy that the air-filled helmet has passed the EN1078 tests, which subjects helmets to harsher vertical impact conditions that use higher vertical impact speeds and kerbstone anvils. Other studies have shown that other air-filled helmets, such as Hövding 3.0 and Bumpair, prolong impact duration and reduce PLA to less than 100 g regardless of the impact location^14,20,25,27^. However, the VENETETE helmet features a design that allows it to sit permanently on the cyclist’s head. Additionally, it can be deflated into a thin structure, which enhances its portability.

The effectiveness of the air-filled helmet in mitigating rotational risk was dependent on the impact location. This may be due to the design of the vertical ribs and Rheon padding, which affects head rotation in different impact locations. The flexibility of the vertical ribs under side loading is likely to be the cause for the reduction in PRA, PRV, and BrIC values during pXR impacts, which produce dominant rotation in the coronal plane. However, the rigidity of the ribs under loading in other directions may restrict head motion under pZR impacts, resulting in higher rotational kinematics^44^. This discrepancy in rotational kinematics across impact locations has been observed in other rotation-management technologies. It is shown for instance that helmets fitted with MIPS are more effective in reducing rotational motion in pXR impacts^20^. These results provide designers with targets for improving rotational performance, particularly under pZR impacts.

Mitigation against skull fractures, caused by direct forces, and diffuse brain injuries, caused by rotation, contributes to a helmet’s overall safety performance^6^. Despite the air-filled helmet and the middle-ranked EPS helmet having a similar rotational risk (a difference of only 6.9%), the air-filled helmet offered a 19.9% improvement in overall protection due to its superior linear protection. In comparison, despite the MIPS helmet having worse linear protection than the air-filled helmet, it still resulted in a 32.3% improvement in overall risk protection due to its equally good linear and rotational risk protection. Incorporating both injury risks into an overall risk can encourage a more holistic approach to helmet design, which concurrently optimises helmets for protecting against both linear and rotational brain injury mechanisms.

We used different injury criteria to separately predict the risk of skull fractures and diffuse brain injuries. This is because each of these pathologies have distinct mechanisms. Skull fractures, and associated injuries such as extradural haematoma, are produced by direct forces^38,45^. Although different criteria have been proposed for skull fractures, such as head injury criterion (HIC), skull fracture criterion (SCF), peak force and PLA, we decided to use PLA. This is because PLA is used in all helmet standards, and it can predict skull fractures, as shown in previous studies on post-mortem human subjects^46^. Diffuse brain injuries are mainly produced by head rotation^38^, and we used BrIC to predict them. Although other criteria for predicting diffuse brain injuries have been proposed, BrIC is used here because it can be easily calculated and it takes into account the sensitivity of the brain to the direction of head rotation. In addition, a recent study of helmeted headform oblique impacts has shown that BrIC is strongly correlated with brain strain predicted with a range of brain finite element models^42^ .

This study has limitations. The helmets were not subjected to testing at different temperatures as required by EN1078, which stipulates testing at both − 20° C and 50° C^30^. Testing at varying temperatures is especially relevant in a pressurised object like the air-filled helmet, where the internal pressure fluctuates with temperature^47^. Another limitation is that equal weights were given to the linear and rotational risks to calculate the overall risk. These weights may be revised in future depending on the availability of real-world data. Another limitation is related to the samples tested. Due to the limited availability of the air-filled VENTETE helmets, which were still in the prototype phase, we conducted three additional impacts at location pXR using the same helmet, but on the opposite side. This was necessary because the initial impact lacked recorded kinematics data. While impacts at locations 180° apart generally do not affect results for an EPS helmet, their effects on a helmet utilizing air technology is less clear. Our results and previous research show consistent PLA results across consecutive impacts^25^, but further investigation is needed to confirm this for air-filled helmets.

## Conclusions

This study presents the first evaluation of a commercially available air-filled bicycle helmet under oblique impacts, addressing a critical gap in helmet safety research. In summary, we found that the air-filled helmet demonstrated 44.1% reduction in linear risk during oblique impacts, compared with EPS helmets. This reduction is attributed to the air-filled chambers, which prolong impact duration and enhance energy absorption without bottoming out. The effectiveness in rotational protection varied across impact locations, and the air-filled helmet’s overall rotational risk was slightly better than the middle ranked EPS helmet, highlighting areas for design optimisation. These results show the feasibility of using air-filled structures to develop helmets with improved head protection and portability. In addition, such technologies may be used to improve protection of other types of head covering, such as head gear designed for older population and Sikh turbans^48,49^. Future studies should explore enhancements to air-filled structures that improve rotational management while maintaining their linear protection benefits. Additionally, testing under different environmental conditions, such as different temperatures, will be essential to assess performance robustness in real-world cycling scenarios. We hope that this work will encourage the design of more innovative helmets aiming at addressing safety along other user requirements.

## Electronic supplementary material

Below is the link to the electronic supplementary material.

Supplementary Material 1

## Data Availability

Data is provided within the supplementary material.
