# Supplementary material for: Superior linear and comparable rotational protection of an air-filled helmet versus foam helmets
Source: Sci Rep. 2025 Jul 27;15:27364. doi: 10.1038/s41598-025-10615-9 (PMC12301445; doi:10.1038/s41598-025-10615-9)
Supplement: Supplementary file 1 — Supplementary Material 1 [file 41598_2025_10615_MOESM1_ESM.pdf]

Appendix 1

Time-history of all kinematic measurements for all tested helmet models across all impact locations. The dotted line represents the average resultant kinematics. The colour-filled area represents the range between the maximum and minimum values across the three repetitions of each helmet.

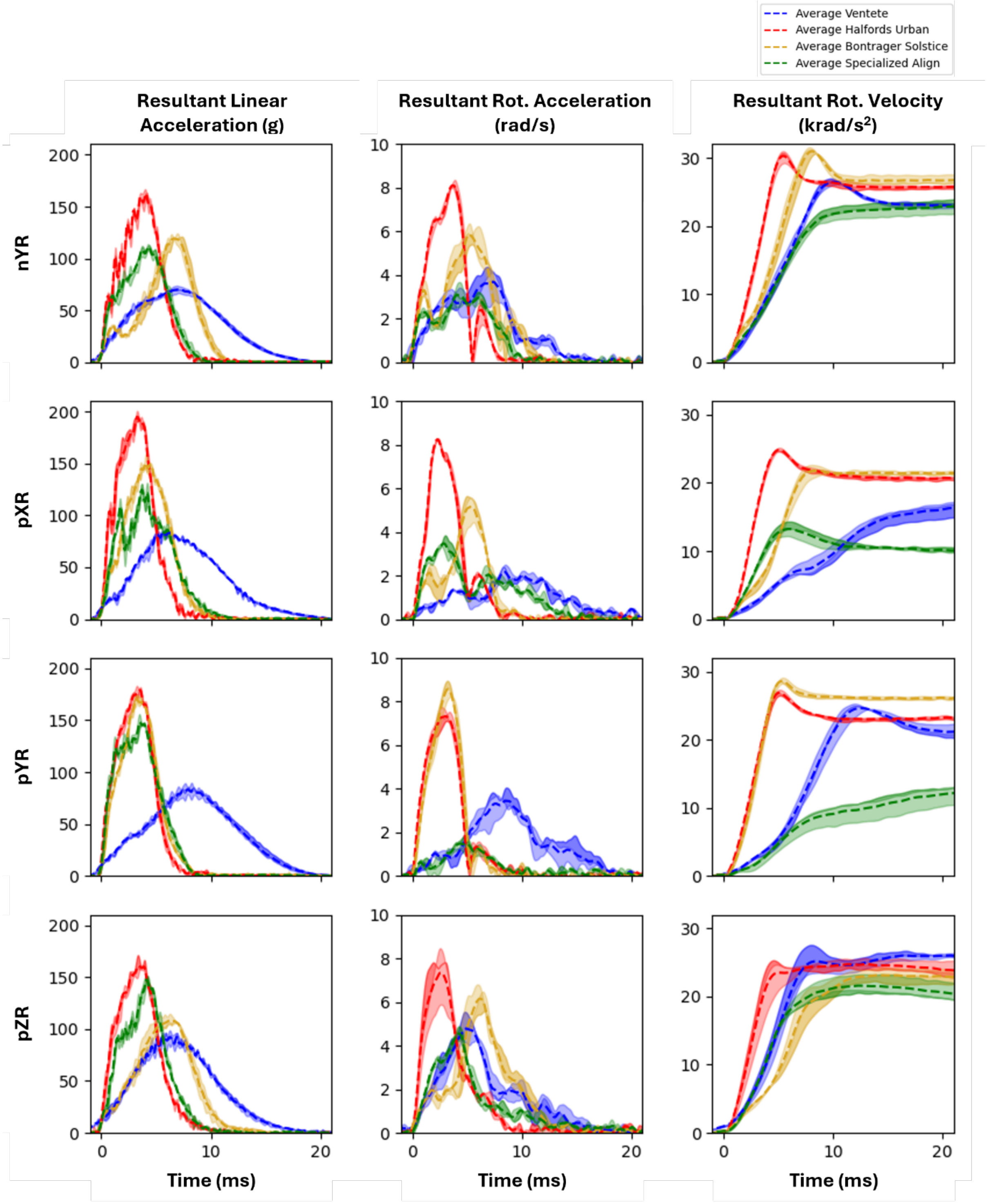

Appendix 2

Kinematic results for all repeats of helmets, in addition to mean, standard deviation, coefficients of variation for the peak linear acceleration (PLA), peak rotational velocity (PRV), peak rotational acceleration (PRA) and the brain injury criterion (BrIC). Non-weighted linear and rotational risk for each impact are also included.

Test ID	Impact	PLA (g)	PRV (rad/s)	PRA (krad/s^2)	BrIC	Non-weighted Linear Risk	Non-weighted Rot Risk
2024-03-25_Ventete_pXR_01	pXR	85.3	17.8	2.5	0.324	0.100	0.158
2024-03-25_Ventete_pXR_02		85.1	15.6	2.4	0.293	0.099	0.121
2024-03-25_Ventete_pXR_03		84.8	18.0	1.9	0.342	0.099	0.182
Average		85.1	17.1	2.3	0.320	0.099	0.154
SD		0.3	1.3	0.3	0.025	0.000	0.031
Coefficient of Variance (%)		3%	8%	14%	8%	0%	20%
2024-03-25_Ventete_pYR_04	pYR	78.9	24.5	3.2	0.446	0.092	0.346
2024-03-25_Ventete_pYR_05		85.9	25.0	4.1	0.479	0.100	0.408
2024-03-25_Ventete_pYR_06		89.8	25.3	3.7	0.465	0.105	0.382
Average		84.9	24.9	3.7	0.463	0.099	0.379
SD		5.5	0.4	0.4	0.017	0.006	0.031
Coefficient of Variance		7%	2%	11%	4%	6%	8%
2024-03-25_Ventete_nYR_04	nYR	66.8	26.1	3.4	0.468	0.080	0.387
2024-03-25_Ventete_nYR_05		70.0	27.0	4.4	0.487	0.083	0.421
2024-03-25_Ventete_nYR_06		73.9	26.5	4.2	0.477	0.087	0.404
Average		70.2	26.5	4.0	0.477	0.083	0.404
SD		3.5	0.5	0.5	0.009	0.004	0.017
Coefficient of Variance		5%	2%	13%	2%	4%	4%
2024-03-25_Ventete_pZR_01	pZR	85.7	26.4	4.9	0.667	0.100	0.737
2024-03-25_Ventete_pZR_02		96.5	27.6	5.6	0.642	0.114	0.699
2024-03-25_Ventete_pZR_03		98.9	25.8	4.3	0.636	0.117	0.690
Average		93.7	26.6	4.9	0.648	0.110	0.709
SD		7.0	0.9	0.6	0.016	0.009	0.025
Coefficient of Variance		8%	3%	12%	2%	8%	4%
2024-07-17_BontragerSol_pXR_01	pXR	159.4	21.4	4.4	0.330	0.226	0.166
2024-07-17_BontragerSol_pXR_02		148.3	21.9	5.6	0.333	0.202	0.169
2024-07-17_BontragerSol_pXR_03		150.4	22.6	5.6	0.349	0.206	0.192
Average		152.7	22.0	5.2	0.337	0.212	0.176
SD		5.9	0.6	0.7	0.010	0.013	0.014
Coefficient of Variance		4%	3%	13%	3%	6%	8%
2024-07-17_BontragerSol_pYR_01	pYR	167.9	29.1	8.6	0.518	0.246	0.479
2024-07-17_BontragerSol_pYR_02		174.5	28.6	9.0	0.505	0.263	0.455
2024-07-17_BontragerSol_pYR_03		170.8	28.2	8.2	0.500	0.254	0.446
Average		171.0	28.6	8.6	0.508	0.254	0.460

SD		3.3	0.4	0.4	0.009	0.008	0.017
Coefficient of Variance		2%	2%	4%	2%	3%	4%
**2024-07-17_BontragerSol_nYR_04**	*nYR*	124.3	31.6	6.2	0.559	0.156	0.556
**2024-07-17_BontragerSol_nYR_05**		121.1	30.6	5.4	0.544	0.150	0.528
**2024-07-17_BontragerSol_nYR_06**		120.2	31.2	6.4	0.556	0.149	0.551
Average		121.9	31.1	6.0	0.553	0.152	0.545
SD		2.1	0.5	0.5	0.008	0.004	0.015
Coefficient of Variance		2%	2%	9%	1%	2%	3%
**2024-07-30_BontragerSol_pZR_04**	*pZR*	106.5	23.3	6.5	0.572	0.128	0.579
**2024-07-30_BontragerSol_pZR_05**		108.3	22.3	5.6	0.536	0.130	0.513
**2024-07-30_BontragerSol_pZR_06**		114.8	24.2	6.8	0.586	0.140	0.604
Average		109.9	23.3	6.3	0.565	0.133	0.565
SD		4.4	1.0	0.6	0.026	0.007	0.047
Coefficient of Variance		4%	4%	10%	5%	5%	8%
**2024_07_17_SpecializedA_pXR_01**	*pXR*	120.3	12.2	3.5	0.252	0.149	0.081
**2024_07_17_SpecializedA_pXR_02**		130.4	14.3	3.8	0.292	0.167	0.120
**2024_07_17_SpecializedA_pXR_03**		131.4	13.3	3.4	0.278	0.168	0.106
Average		127.3	13.3	3.6	0.274	0.161	0.102
SD		6.1	1.1	0.2	0.020	0.011	0.020
Coefficient of Variance		5%	8%	7%	7%	7%	19%
**2024_07_17_SpecializedA_pYR_01**	*pYR*	146.4	13.5	1.7	0.237	0.198	0.068
**2024_07_17_SpecializedA_pYR_02**		148.2	13.3	1.8	0.236	0.202	0.067
**2024_07_17_SpecializedA_pYR_03**		156.1	10.9	1.2	0.193	0.219	0.039
Average		150.2	12.6	1.6	0.222	0.206	0.058
SD		5.1	1.4	0.3	0.025	0.011	0.017
Coefficient of Variance		3%	11%	19%	11%	5%	29%
**2024_07_17_SpecializedA_nYR_04**	*nYR*	113.9	22.4	3.7	0.398	0.139	0.265
**2024_07_17_SpecializedA_nYR_05**		112.7	24.5	3.2	0.434	0.137	0.326
**2024_07_17_SpecializedA_nYR_06**		107.7	23.6	2.8	0.417	0.129	0.298
Average		111.4	23.5	3.2	0.416	0.135	0.296
SD		3.3	1.0	0.4	0.018	0.005	0.031
Coefficient of Variance		3%	4%	14%	4%	4%	10%
**2024_07_17_SpecializedA_pZR_04**	*pZR*	143.5	23.3	4.5	0.465	0.192	0.381
**2024_07_17_SpecializedA_pZR_05**		151.3	20.6	4.9	0.424	0.208	0.309
**2024_07_17_SpecializedA_pZR_06**		150.6	21.0	4.2	0.433	0.207	0.324
Average		148.5	21.6	4.6	0.440	0.202	0.338
SD		4.3	1.5	0.3	0.022	0.009	0.038
Coefficient of Variance		3%	7%	7%	5%	4%	11%
**2023-02-13_HalfordsUrban_pXR_01**	*pXR*	194.3	24.8	8.3	0.390	0.316	0.253
**2023-02-13_HalfordsUrban_pXR_02**		193.1	24.6	8.26	0.378	0.313	0.234
**2023-02-13_HalfordsUrban_pXR_03**		200.8	25.2	8.17	0.395	0.335	0.261
Average		196.1	24.7	8.24	0.388	0.321	0.250

SD		4.14	0.31	0.067	0.009	0.012	0.014
Coefficient of Variance		2%	1%	1%	2%	4%	6%
**2023-02-13_HalfordsUrban_pYR_01**	*pYR*	183.2	26.9	7.75	0.475	0.286	0.400
**2023-02-13_HalfordsUrban_pYR_02**		180.2	26.5	7.11	0.469	0.278	0.389
**2023-02-13_HalfordsUrban_pYR_03**		175.1	27.3	7.53	0.484	0.264	0.416
Average		179.5	26.9	7.46	0.476	0.276	0.402
SD		4.1	0.4	0.33	0.008	0.011	0.014
Coefficient of Variance		2%	1%	4%	2%	4%	3%
**2024-02-27_HalfordsUrban_nYR_04**	*nYR*	166.9	29.2	8.02	0.518	0.244	0.478
**2024-02-27_HalfordsUrban_nYR_05**		163.5	30.7	8.09	0.543	0.236	0.526
**2024-02-27_HalfordsUrban_nYR_06**		158.4	30.99	8.37	0.549	0.224	0.537
Average		162.9	30.3	8.16	0.536	0.235	0.514
SD		4.28	0.96	0.19	0.017	0.010	0.031
Coefficient of Variance		3%	3%	2%	3%	4%	6%
**2024-02-28_HalfordsUrban_pZR_04**	*pZR*	158.2	25.7	5.88	0.584	0.224	0.601
**2024-02-28_HalfordsUrban_pZR_05**		171.2	25.3	7.83	0.588	0.255	0.608
**2023-02-28_HalfordsUrban_pZR_06**		161.6	24.7	8.47	0.596	0.231	0.622
Average		163.7	25.2	7.39	0.590	0.237	0.610
SD		6.74	0.5	1.35	0.006	0.016	0.011
Coefficient of Variance		4%	2%	18%	1%	7%	2%
